# Life with chronic pain during COVID-19 lockdown: the case of patients with small fibre neuropathy and chronic migraine

**DOI:** 10.1007/s10072-020-04890-9

**Published:** 2020-11-17

**Authors:** Monica Consonni, Alessandra Telesca, Licia Grazzi, Daniele Cazzato, Giuseppe Lauria

**Affiliations:** 1 Neuroalgology Unit, Fondazione IRCCS Istituto Neurologico Carlo Besta, Via Celoria 11, 20133 Milan, Italy; 2 Neurophysiology Unit, Fondazione IRCCS Istituto Neurologico Carlo Besta, Milan, Italy; 3 Department of Biomedical and Clinical Sciences “Luigi Sacco”, University of Milan, Milan, Italy

**Keywords:** COVID-19 distress, Chronic migraine, Small fibre neuropathy, Catastrophism, Quality of life, Chronic pain

## Abstract

**Objective:**

We aimed at investigating the impact of COVID-19-related distress on patients with chronic pain, highlighting the effects of changes in individual habits and public health care reconfiguration on physical and psychological health.

**Methods:**

During the pandemic, 80 participants (25 patients with small fibre neuropathy (SFN), 42 patients with chronic migraine (CM) and 13 patients’ healthy family members (HFM)) were asked to evaluate their COVID-19 complains, changes in habits and clinical management, behaviour, mood, loneliness, quality of life (QoL), physical and mental health and coping strategies. Data were analysed by Spearman rho correlations and Mann-Whitney *U* tests.

**Results:**

Patients had lower QoL, lower physical health and higher catastrophizing attitude towards pain than HFM. During the pandemic, SFN patients referred greater decline in clinical symptoms, worries about contagion and discomfort for disease management changes than CM patients. In the SFN group, the higher levels of disability were associated with suffering from changes in neurologist-patient relationship. CM patients complained of agitation/anxiety that was related to feelings of loneliness, depressive mood and catastrophism.

**Discussion:**

Despite similar complains of change in habits and worries about COVID-19 pandemic, SFN and CM patients had distinct reactions. In SFN patients, pandemic distress impacted on physical health with worsening of clinical conditions, especially suffering from changes in their care. In CM patients, pandemic distress affected behaviour, mainly with psychological frailty. This suggests the need to customize public health care for patients with distinct chronic pain conditions.

## Introduction

The outbreak of the new coronavirus pneumonia (COVID-19) turned to become a sudden public health crisis that strongly influenced psychological and physical health of the general population [1, 2]. Across the world, people who have potentially come into contact with the infection were asked to stay at home or in dedicated quarantine facilities. Reports concerning the psychological effects of self-isolation during past epidemics and pandemics (e.g. SARS, Ebola) confirmed that quarantined people had higher level of distress including posttraumatic stress disorder and depression [3, 4].

In Italy, the lockdown imposed between March 8th and May 3rd 2020 included restrictions of general population movement except for health care personnel and workers employed in social relevant activities and for urgent health/work needs. This stopped all elective and routine casework while healthcare providers have had to change their practice significantly. Patients with chronic diseases suffered from major burden of this sudden social reorganization due to the need of frequent access to health facilities, including in-person visits, follow ups and treatments [5, 6]. In our tertiary centre in the Lombardy region, in the most exposed area to the exponential contagion of Northern Italy, the lockdown modified dramatically our regular clinical practice of managing patients with chronic pain disorders, with increasing discomfort of those followed for chronic neuropathic pain (NP) and chronic migraine (CM) [7]. Accordingly, individuals living in Northern Italy felt the health emergency as more urgent than what individuals living in Central and Southern regions did [8].

Chronic pain is a common condition worldwide and is frequently associated with decreased health-related quality of life and high levels of psychological distress [9]. NP incidence is about 7–10% of the general population [10] and CM at least 2% [11]. Both these conditions are characterized by high levels of disability and impact several life aspects, such as emotion, work and social life [12–14]. These patients need to be carefully followed to arrange their therapies. Due to the lockdown measures imposed by the Italian government, NP and CM patients were forced to change their clinical management: scheduled visits were cancelled, in-person check-up appointments postponed, therapeutic sessions gone missing, while telemedicine was rapidly introduced in clinical practice. This unavoidable condition likely contributed to increase a feeling of missing and abandoning, possibly worsening pain considering its strong relationship between with psychological fragility [5].

Our study aimed at evaluating whether and to what extent the changes occurring during the COVID-19 healthy emergency in the clinical status, clinical management, habits and behaviour influenced mood, loneliness, coping strategies and attitude to catastrophism in patients suffering from two different chronic pain conditions: small fibre neuropathy (SFN) and chronic migraine (CM).

## Methods

### Participants

Between May 2nd and June 11st 2020, patients were enrolled from the out-patient service if they had clinical and laboratory-supported diagnosis of SFN [15] and NRS > 0 at enrolment or diagnosis of CM according to international criteria [11]. They were asked to participate in the study in the context of in-person (34%) telemedicine (28%) follow-up or by phone for those whose visit was scheduled after June 15th 2020 (38%). Healthy family members (HFM) were also enrolled as healthy controls. The HFM group was included to verify the impact of COVID-19 lockdown in a pain-free healthy sample. Inclusion criteria for all participants were age > 18 years and written informed consent. Exclusion criteria included any clinical condition affecting the ability to complete self-administered psychometric scales. HFM had no diagnosis of neurological or psychiatric disorder. Informed consent was obtained from each subject. The study is in accordance with the ethical standards of our responsible institutional committee on human experimentation and with the Helsinki Declaration of 1975, as revised in 1983.

### Materials

Participants were asked to complete a newly developed questionnaire assessing psychological distress due to COVID-19 [16]. The COVID-19 distress questionnaire was provided by paper or digital format or administered through a phone call. The questionnaire evaluates (a) the perceived risk to contract COVID-19, (b) concerns about COVID-19, (c) changes in clinical management, (d) changes in everyday habits due to the lockdown and (e) changes in behaviour due to COVID-19 emergency. All participants were also asked to provide psychometric scales addressing mood (Hospital Anxiety and Depression Scale[17]), loneliness [18], coping strategies (pain catastrophizing scale, PCS [19]; coping strategies questionnaire, CSQ [20]) and the health status (12-Item Short Form Survey, SF-12 [21]). Additionally, patients compiled specific disease-related questionnaires assessing pain and quality of life (EUROHIS-QOL) [22]. These questionnaires have been modified in order to have the patients answering the questions in reference to a period before COVID-19 pandemic (December 2019 to January 2020) and to the study period (May–June 2020). Specifically, SFN patients were asked (i) to judge how sick they feel using a 7-point Likert scale, from 1 = normal to 7 = most sick (global impression); (ii) to evaluate, on average, how strong was the experienced pain using a 10-point Likert scales, from 0 = no pain to 10 = worst pain (Numeric Rating Scale—NRS); (iii) to specify the occurrence of the following symptoms (maximum 7): burning feet, painful cold, electric shock, tingling, sting like pins, numbness, itching, feeling of discomfort when rubbing and restless legs; (iv) to specify the occurrence of the following autonomic symptoms: sweating, diarrhoea, constipation, urinary disorders, dry eyes, dry mouth, dizziness, episodes of sudden heat and/or redness; (v) to describe the course of pain over the specified period selecting one of the following descriptions: 1 = persistent with minor fluctuations; 2 = persistent with major pain attacks; 3 = accesses of pain without substantial underlying pain; 4 = accesses of pain with constant underlying pain. CM completed the headache impact test (HIT-6) [23] considering the two time frames.

### Statistical analyses

Kruskal-Wallis test was used to analyse between-group differences of demographical and psychometric variables listed in Table 1. Variables surviving Bonferroni correction (*p* < 0.003) were included in post hoc analyses (Mann-Whitney *U* test) to assay differences between patient subgroups and HFM. The Mood’s median tests for between-group differences in psychological distress due to COVID-19 pandemic measured with the newly developed questionnaire listed in Table 2, in the view of the explorative purpose, were considered significant for *p* value lower than 0.01 and Mann-Whitney *U* post hoc tests were performed accordingly. Wilcoxon signed rank tests were used to analyse changes in clinical features and QoL within groups (Table 3). Spearman rho correlation analyses were performed to explore the relationship between chronic pain condition and mood (HADS scores), QoL, coping strategies (CSQ and PCS subscales) and physical and mental health (SF12 subscales). Analyses were performed separately for SFN and CM patients (Tables 4). Additionally, Spearman rho correlation analyses explored the association between COVID-19 pandemic impact and clinical and neuropsychological data of SFN and CM patients. To adjust for multiple comparisons, we considered only correlations with *p* value ≤ 0.003. IBM SPSS Statistics (version 21) was used to perform analyses.

**Table 1:** Demographical and psychometric data of patients with small fibre neuropathy (SFN) and chronic migraine (CM) and healthy volunteers. Significant between-group differences are reported

	No.	SFN patients	No.	CM patients	No.	Healthy volunteers	Sig.
Age	25	55.84 ± 13.1	42	49.00 ± 10.3	13	52.67 ± 17.3	n.s.
Sex (M/F)	25	16/9	42	3/39	13	9/4	< 0.001^a,c^
Education (years)	25	12.48 ± 3.61	42	14.08 ± 2.9	13	14.85 ± 3.4	n.s.
HADS-Anxiety	25	8.45 ± 4.9	40	7.17 ± 4.2	13	5.00 ± 2.5	n.s.
HADS-Depression	25	5.87 ± 4.3	40	4.65 ± 3.5	13	3.00 ± 2.3	n.s.
QoL	25	22.39 ± 6.3	39	26.82 ± 4.6	13	29.92 ± 3.7	0.011^a^; < 0.001^b^; 0.041^c^
Physical health (SF-12)	24	32.07 ± 9.7	41	40.09 ± 8.0	12	53.30 ± 4.1	< 0.001^a,b,c^
Mental health (SF-12)	24	44.17 ± 11.9	41	45.02 ± 12.0	12	52.51 ± 6.1	n.s.
Coping strategies (CSQ)							
CSQ Distraction	24	0.50 ± 0.3	41	0.44 ± 0.3	12	0.64 ± 0.2	n.s.
CSQ Ignore	24	0.46 ± 0.2	41	0.41 ± 0.2	12	0.43 ± 0.2	n.s.
CSQ Self-determination	24	0.70 ± 0.2	41	0.69 ± 0.2	12	0.61 ± 0.2	n.s.
CSQ Distance	24	0.19 ± 0.2	41	0.20 ± 0.2	11	0.19 ± 0.2	n.s.
CSQ Catastrophism	24	0.45 ± 0.2	41	0.41 ± 0.2	11	0.16 ± 0.1	< 0.001^b^; 0.002^c^
CSQ Prayer	24	0.34 ± 0.3	41	0.50 ± 0.3	11	0.33 ± 0.3	n.s.
Catastrophism tot (PCS)	24	0.65 ± 0.3	39	0.47 ± 0.2	12	0.27 ± 0.1	0.010^a^; < 0.001^b^; 0.003^c^
PCS helplessness	24	0.62 ± 0.3	39	0.43 ± 0.2	12	0.21 ± 0.1	0.018^a^; < 0.001^b^; 0.002^c^
PCS rumination	24	0.72 ± 0.3	39	0.60 ± 0.2	12	0.38 ± 0.2	0.001^b^; 0.009^c^
PCS magnification	24	0.57 ± 0.3	39	0.31 ± 0.2	12	0.17 ± 0.2	0.002^a^; < 0.001^b^

*CSQ* coping strategy questionnaire, *HADS* Hospital Anxiety and Depression Scale, *PCS* pain catastrophizing scale, *QoL* quality of life, *SF-12* 12-Item Short Form Survey

^a^SFN vs. CM

^b^SFN vs. HFM

^c^CM vs. HFM

**Table 2:** Evaluation of the impact of COVID-19 on SFN disease management and daily life in patients with small fiber neuropathy (SFN) and chronic migraine (CM) and in healthy family members (HFM). Results are presented as median (range); mean (standard deviation).

	SFN patients *N* = 25	CM patients *N* = 42	HFM *N* = 13	Group differences
COVID-19 Questionnaire (A–E)				
A. Perceived risk of COVID-19				
1 How much are you able to avoid COVID-19?^a^	3 (1–5); 3.37 (0.9)	3 (1–5); 2.70 (1.2)	3 (1–5); 3.00 (1.2)	n.s.
2 # information sources on COVID-19 (max 8)	5 (1–7); 4.58 (1.3)	4.5 (1–8); 4.5 (1.8)	4 (0–6); 3.76 (1.8)	n.s.
3 # actions taken to avoid contagion (max 11)	11 (6–11); 10.20 (1.4)	11 (6–12); 10.36 (1.2)	11 (7–11); 10.08 (1.3)	n.s.
B. Concern about COVID-19				
1 worries my illness make me more fragile in case of infection^a^	4 (1–5); 3.30 (1.2)	2 (1–5); 2.17 (1.1)	1 (1–3); 1.14 (0.8)	< 0.001^b,c,d^
2 Worries in the event of an infection^a^	3.5 (1–5); 3.25 (1.2)	3 (1–5); 2.92 (0.9)	2 (1–4); 2.15 (1.1)	0.012^c^
3 Thinking of COVID-19^a^	3 (2–5); 2.91 (0.8)	3 (1–5); 3.12 (0.9)	3 (1–5); 2.61 (1.0)	n.s.
4 Thinking that COVID-19 can worry my family^a^	4 (2–5); 3.58 (0.9)	3 (1–5); 3.45 (1.0)	3 (1–5); 3.00 (1.2)	n.s.
C. Change in disease management				
1 Drug management change^a^	1 (1–5); 1.58 (1.2)	1 (1–4); 1.70 (1.0)	n.a.	n.s.
2 Change in neurologist-patient relationship^a^	2 (1–5); 2.63 (1.7)	1 (1–5); 2.04 (1.0)	n.a.	n.s.
3 Feelings of being forgotten/rejected by clinicians^a^	1 (1–5); 2.52 (1.8)	1 (1–5); 1.63 (1.3)	n.a.	< 0.001^b^
4 Concern about negative consequences of COVID-19 healthy emergency on the management of the disease by clinicians^a^	2 (1–5); 2.50 (1.5)	1 (1–5); 1.78 (1.1)	n.a.	< 0.001^b^
D. Change in habits due to COVID-19 state of emergency				
1 Out-of-home habits^a^	4 (1–5); 3.58 (1.1)	4 (1–5); 3.83 (1.0)	3 (2–5); 3.84 (1.1)	n.s.
2 Household habits^a^	3 (1–5); 2.87 (1.3)	3 (1–5); 2.97 (1.2)	3 (1–5); 2.93 (1.4)	n.s.
3 Use of social networks^a^	3 (1–5); 3.12 (1.3)	3 (1–5); 2.83 (1.3)	2 (1–5); 2.65 (1.4)	n.s.
4 Work/retirement^a^	4 (1–5); 3.62 (1.4)	4 (1–5); 3.48 (1.4)	3 (1–5); 3.15 (1.6)	n.s.
5 Personal care^a^	2 (1–5); 2.60 (1.4)	2.5 (1–5); 2.61 (1.3)	2 (1–4); 2.41 (1.4)	n.s.
E. Change in behaviour due to COVID-19 state of emergency				
1 Irritable/nervous^a^	1.5 (1–4); 1.66 (0.8)	2 (1–5); 2.09 (1.0)	2 (1–4); 2.23 (1.0)	n.s.
2 Agitated/anxious^a^	2 (1–5); 1.95 (1.0)	3 (1–5); 2.21 (1.0)	2 (1–3); 1.76 (0.7)	0.018^b^; 0.013^d^
3 Sad/depressed^a^	1.5 (1–4); 1.83 (0.9)	2 (1–5); 1.86 (0.9)	1 (1–3); 1.38 (0.6)	n.s.
4 Bored^a^	2 (1–5); 2.25 (1.2)	1.5 (1–5); 2.09 (1.1)	2 (1–5); 2.23 (1.2)	n.s.
5 Increased consumption of alcohol/cigarettes^a^	1 (1–3); 1.16 (0.5)	1 (1–5); 1.14 (0.4)	1 (1–2); 1.07 (0.3)	n.s.

*n.s.* not significant differences

^a^The range of responses varied from 1 (not at all) to 5 (extremely)

^b^SFN vs. CM

^c^SFN vs. HFM

^d^CM vs. HFM

**Table 3:** Clinical data of patients with small fibre neuropathy (SFN) and chronic migraine (CM) and in healthy family members (HFM) before and during COVID-19 pandemic

	No.	December 2019 to January 2020	No.	May–June 2020	Sig.
SFN					
GI (min 1; max 7)	24	3.96 ± 1.45 (1–6)	24	4.00 ± 1.8 (2–7)	n.s.
NRS (min 1; max 10)	24	6.45 ± 2.16 (3–10)	23	7.13 ± 2.2 (3–10)	0.013
Pain symptoms (min 0; max 7)	24	4.08 ± 1.8 (1–7)	24	4.58 ± 1.8 (2–7)	0.047
Autonomic symptoms (min 0; max 12)	25	5.52 ± 3.2 (0–11)	25	5.72 ± 3.3 (0–11)	n.s.
Pain course (min 1; max 4)	24	2.7 ± 1.0 (1–4)	23	3.21 ± 0.9 (1–4)	0.046
1. Persistent with minor fluctuations	3	12%	1	4%	-
2. Persistent with major attacks	9	38%	6	25%	-
3. Pain accesses without substantial underlying pain	4	17%	5	21%	-
4. Pain accesses with constant underlying pain	8	33%	12	50%	-
QoL	23	23.7 ± 5.2	23	22.39 ± 6.3	n.s.
CM					
HIT total score	42	64.09 ± 7.0	42	62.28 ± 6.2	n.s.
Little-no/some/substantial/severe	42	2 / 1 / 5 / 34	42	2 / 2 / 9 / 29	n.s.
QoL	39	28.66 ± 4.3	41	26.82 ± 4.6	0.037
HFM					
QoL	13	32.53 ± 2.7	13	29.92 ± 3.7	0.013

Data are expressed as mean ± standard deviants (range)

*GC* global impression, *NRS* Numeric Rating Scale, *QoL* quality of life

**Table 4:**
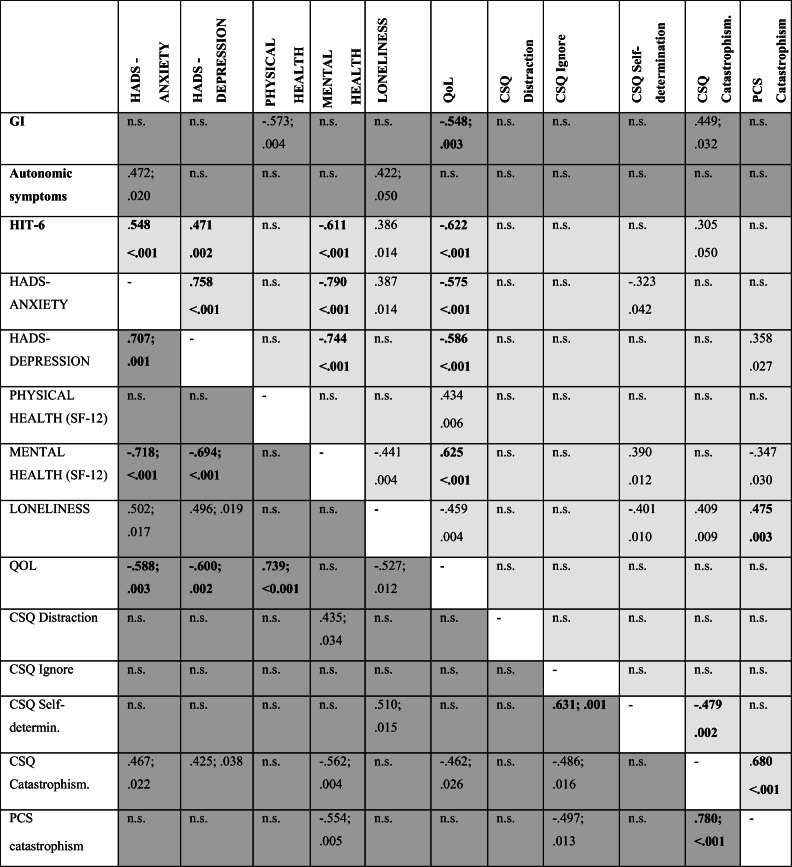
Correlation analyses of clinical and neuropsychological data of patients with small fibre neuropathy (SFN; grey) and chronic migraine (CM, light grey). Correlation surviving Bonferroni correction are reported in bold

No significant correlations were found for CSQ Distance subscale, CSQ Prayer subscale, Pain-relates symptoms scale and NRS; these variables are not listed in the table

*CSQ* coping strategies questionnaire, *GI* global impression, *HADS* Hospital Anxiety and Depression Scale, *HIT-6* headache impact test-6 Items, *n.s* not significant (*p* > 0.05), *PCS* pain catastrophizing scale, *QoL* quality of life, *SF-12* 12-Item Short Form Survey

## Results

Twenty-five SFN patients, 42 CM patients and 13 HFM volunteers participated in the study completed the COVID-19 distress questionnaire (Table 2). Twenty-three SFN patients filled out questionnaires by e-mails and two SFN patients were contacted by phone to fulfil questionnaires. None of the SFN had planned telemedicine or in-person visits between study period observation (2 May–11 June 2020). Twenty-one CM filled out questionnaires during in-person visits, whereas 21 CM patients filled out questionnaires by e-mails. All HFM completed the questionnaires by e-mails. Patients and HFM groups had similar age and education level. As expected, based on epidemiological data, there were more females in the CM group than in the others (*p* < 0.001). The number of participants fulfilling scales and questionnaires are reported in Table 1 and in Table 3. Between-group analyses showed a significant difference in physical health (*X*^2^ = 31.313, *p* < 0.001), QoL (*X*^2^ = 14.778, *p* = 0.001) and coping strategies, namely catastrophism (CSQ: *X*^2^ = 12.731, *p* = 0.002; PCS total score: *X*^2^ = 17.425, *p* < 0.001; PCS helplessness: *X*^2^ = 16.307; PCS rumination: *X*^2^ = 12.420, *p* = 0.002; PCS magnification: *X*^2^ = 16.922, *p* < 0.001). As expected, post hoc analyses documented that SFN and CM patients had lower QoL, lower scores on scale assessing physical health (SF12) and higher attitude towards dysfunctional coping strategies than HFM. The SFN group reported the most severe scores (Table 1). Accordingly, SFN group differed also on measures of distress related to COVID-19 pandemic (Table 2), with patients were more worried to get infected (item B1: *X*^2^ = 12.550, *p* = 0.002; item B2: *X*^2^ = 9.829, *p* = 0.007) and referring more changes in behaviour (agitation/anxiety *X*^2^ = 9.332, *p* = 0.009). Post hoc analyses confirmed that SFN patients were more worried about COVID-19 infection and reported greater changes in disease management due to COVID-19 healthy emergencies than CM patients. CM patients reported greater change in behaviour and felt overall more agitated and anxious than before the pandemic (Table 2). Within-group comparisons documented worsening of clinical symptoms in SFN patients and reduced QoL in CM and HFM groups (*p* < 0.05; Table 3).

Results of correlation analyses between clinical and psychometric variables are reported in Table 4. Briefly, in the SFN group, the greater the complaining of disease, the lower the QoL that was directly associated to anxiety and depression, and inversely related to physical health. In the CM group, adverse headache impacts were associated with depression, anxiety and reduced global mental health and QoL. Of note, even not surviving to Bonferroni corrections, catastrophism was the only coping strategy associated with the most severe clinical manifestations both in SFN and CM. Correlation analyses also showed that the COVID-19 pandemic distress, measured with the questionnaire displayed in Table 2, was associated to psychological status and coping strategies. Specifically, in the SFN group, the greater the levels of physical disability (physical health subscale of the SF-12), the greater the suffering from changes in neurologist-patient relationship (item C2; rho = 0.712, *p* < 0.001). In this group, catastrophism (PCS global score) was related to changes in behaviour (sum of items of scale E) during the pandemic (rho = 0.634; *p* = 0.001). In the CM group, patients suffering from changes in clinical management (item C4) were those with lower mental stability (mental health subscale of the SF-12: rho = − 0.475; *p* = 0.003). In the CM group, the greater the changes of behaviour during the pandemic (sum of items of scale E), the higher feeling of loneliness (rho = 0.481; *p* = 0.002) and catastrophism (PCS global score: rho = 0.528; *p* = 0.001). Overall, in all patients, behavioural changes due to COVID-19 lockdown (sum of items of scale E) were associated with mental health (SF-12 subscale; SFN: rho = − 0.608; *p* = 0.002; CM: rho = − 0.494; *p* = 0.001).

## Discussion

We sought to address the impact of the consequences of the lockdown adopted to mitigate the spread of COVID-19 and the reconfiguration of the public health care system on physical and mental health of subjects with chronic pain due to two different diseases. This approach allowed at providing information both on the condition of suffering from chronic pain and on features peculiar of each of the diseases.

The perceived risk of COVID-19 was measured considering the amount of information sources and actions taken to avoid contagion. It was similar in all participants, suggesting that they were informed about the risk of the infection and addressed the Italian government dispositions. The lockdown caused moderate to extreme changes in out-of-home habits, work and household management in half of the participants (Table 2), with a negative impact on QoL of HFM and CM patients [24] and worsening of clinical condition in SFN patients (Table 3). Patients complained also changes in clinical management, including the patient-neurologist relationship and the management of pharmacological therapy (Table 2). The stress-related psychosocial impact of the lockdown is evident and multifaceted. Similarly to our results, several recent studies documented its negative impact on QoL, habits, behaviour and mental health in patients with chronic diseases and in the general population [8, 16, 25, 26]. But we further showed that changes in habits per se did not have any apparent association with psychological well-being, whereas changes in behaviour (e.g. irritability, anxiety, sadness) impacted on patients’ mental health. In our clinical samples, behavioural changes due to lockdown were highly associated with a dysfunctional attitude toward negative self-statement and excessively negative beliefs about the future, namely catastrophism. This finding was in keeping with previous studies showing that patients with chronic pain use passive strategies and catastrophic appraisals [27]. Specifically, during pandemic, catastrophism was a distinctive appraisal of both SFN and CM patients, but it was a massively recurring attitude of the SFN group (Table 1). It is indeed acknowledged that chronic pain is a burdensome condition [13, 28] where anxiety, depression and fatigue can influence patients’ life expectations [29] and QoL [30]. Consistently, in our cohorts, low QoL in SFN and CM patients (Table 4) was associated with anxiety, depression and reduced well-being [31].

Despite similar complains of change in habits and worries about COVID-19 pandemic, SFN and CM patients had distinct reactions. More than half of CM patients were more anxious/agitated than before the pandemic. Behavioural changes affected mainly CM patients with psychological frailty showing dysfunctional attitude toward negative self-statement and excessively negative beliefs about the future, depressive mood and feeling of loneliness. This is consistent with the findings that, overall, CM affects mental health (Table 4) and it can cause depression and anxiety [32, 33]. Furthermore CM patients often have comorbid psychiatric conditions [34], but anxiety disorders particularly exacerbate pain, hypervigilance and the tendency to catastrophize in chronic conditions [32, 33]. Stress and maladaptive coping strategies were found to be major determinants of anxiety [33]. Our findings suggest that underlying dysfunctional social-cognitive mechanisms, exacerbated by COVID-19 pandemic, could have had an impact on coping reactions to chronic pain in CM. In other terms, having positive thinking attitudes and more people in our own social network increases the probability to cope better with COVID-19 pandemic distress. This is in line with the findings showing that, in people with migraine, greater openness to experiences, acceptance and behavioural disengagement are associated with lower impact and disability [35].

SFN patients complained instead of a decline in clinical conditions during pandemic (Table 2), complaining augment in the intensity and frequency of their pain. It might have been an indirect and negative consequence of the changes in clinical management. As correlation analyses suggested, in SFN, the higher the physical health measured with the SF-12, the higher the perceived changes of patients-neurological relationships. SFN patients complained more about possible negative consequence of contagion on their illness than CM patients. This in line with the view that the effect of the pandemic on pain burden may be differentially distributed across and within clinical cohorts, depending on their characteristics [5]. Consistently, SFN, more than CM, suffered from physical disability that affected QoL (Table 4).

During the lockdown from March 8th to May 3rd, chronic pain patients have suffered from changes in clinical management [7]: missed visits, missed therapeutic sessions and rescheduling of their visits. This situation favoured a sense of missing and abandoning that increased the psychological discomfort, especially in SFN patients (Table 2). It is acknowledged that when people with chronic pain are denied assessment and treatment, their condition can worsen significantly [5]. People waiting for assessment often report severe levels of pain that interfere with their ability to function and experience deteriorating health-related QoL and increased depression [36].

Our study has some limitations. Further psychological distress instruments on COVID-19 recently developed [37, 38] could provide more detailed measures of mental health. Compared with face-to-face interviews, online-based self-reporting survey has certain limitations. Lastly, in this cross-sectional study, the survey was conducted between May and June 2020, during a relative weakening of the epidemic in Italy, and changes in QoL and mood drop off over time.

In sum, this study evaluated the impact of changes occurring during the COVID-19 healthy emergency on clinical status, clinical management, habits, behaviour, mood, QoL and coping strategies of patients with SFN and CM. We found that SFN and CM patients had similar complains of change in habits and worries about contagion, but COVID-19-related distress had distinct impact on them. It interfered with physical health in SFN patients and with mental health in CM patients. The opportunity to investigate two different chronic pain conditions revealed that the clinical peculiarities are associated with underlining different psychological status. Even if wider samples of patients with chronic pain would have led to more generalizable results, this evidence should be taken into account and strengthens the need to customize the health care system for chronic pain conditions.

## Data Availability

Readers seeking access to the data should contact the corresponding author. Access will be granted to named individuals in accordance with ethical procedures governing the reuse of sensitive data. Specifically, to obtain the data, requestors must complete a formal data sharing agreement.
